# Structural and functional properties of collagen isolated from lumpfish and starfish using isoelectric precipitation vs salting out

**DOI:** 10.1016/j.fochx.2023.100646

**Published:** 2023-03-15

**Authors:** Naveen Kumar Vate, Przemyslaw Strachowski, Ingrid Undeland, Mehdi Abdollahi

**Affiliations:** a Department of Life Sciences – Food and Nutrition Science, Chalmers University of Technology, 412 96 Gothenburg, Sweden; b Department of Fish Processing Technology, School of Fisheries, Centurion University of Technology and Management, Paralakhemundi, Odisha 761211, India

**Keywords:** Marine collagen, Lumpfish, Starfish, Isoelectric precipitation, Fibril formation

## Abstract

The possibility of replacing the very time and resource demanding salting out (SO) method with isoelectric precipitation (IP) during collagen extraction from common starfish and lumpfish was investigated. The effect of IP on yield, structural and functional properties of the collagens was therefore compared with SO. Application of IP resulted in a higher or similar collagen mass yield compared with SO from starfish and lumpfish, respectively. However, the purity of collagens recovered with IP was lower than those recovered with SO. Replacing SO with IP did not affect polypeptide pattern and tropohelical structural integrity of collagen from the two resources as revealed with SDS-PAGE and FTIR analysis. Thermal stability and fibril formation capacity of collagens recovered with IP were also well preserved. Overall, the results showed that the IP can be a promising resource smart alternative for the classic SO precipitation during collagen extraction from marine resources.

## Highlights

• Collagen was extracted from starfish and lumpfish by a resource smart approach.
• Resource demanding salting-out was replaced by isoelectric precipitation.
• Mass yield and recovery increased by applying isoelectric precipitation.
• Isoelectric precipitation had least effect on structural and functional properties.

## 1 Introduction

Marine collagen is gaining increasing interest as it is having many benefits compared to mammalian collagen. The use of collagen from bovine and porcine sources has religious restrictions (Chakka, Muhammed, Sakhare, & Bhaskar, 2017). Also, bovine collagen has a danger of carrying diseases like bovine spongiform encephalopathy, which has created interest in finding safer alternative sources of collagen (Venien & Levieux, 2005). Today, marine collagen is primarily extracted from fish skin, scales, or bones. However, there is a growing interest for exploring other marine sources for the extraction of collagen including sea urchin wastes, small fish and by-catch or invasive species namely jellyfish, starfish and sponges (Coppola et al., 2020).

Regardless of the source, the collagen is currently isolated using acetic acid, without or with the aid of pepsin enzyme. These extraction processes require several steps of pretreatment and also a final recovery step. The major drawback of these methods is that they are complex, time consuming (5–8 days), requiring huge amounts of resources and yet resulting in low yield. These challenges increase with increasing the complexity of the start material for example when using a whole animal biomass or bony tissues for collagen extraction. Because of all these constraints the isolation of collagen from aquatic resources has become inefficient, expensive and unsustainable (Pal & Suresh, 2016). We have recently shown that the amount of chemical and processing time needed in the pretreatment step of collagen extraction can be substantially reduced using selected assistant technologies (Vate, Undeland, & Abdollahi, 2022). However, still the recovery step of the process in which extracted collagen is precipitated out of the acetic acid at a very high ionic strength induced by addition of salt (called salting out – SO) remains very resource demanding (Liu et al., 2012, Sinthusamran et al., 2013). After the salt-induced precipitation, there is a need for desalting through dialysis in order to remove the residual salts carried along with the extracted collagen before going for lyophilization. This takes up to 3–4 days and requires large volumes of water and a very high centrifugation speed.

Similar to many other proteins, native collagen has a pH-dependent solubility where it shows high solubility at acidic condition which is used for its extraction but low solubility at neutral to alkaline pH values. Utilizing the isoelectric point (pI) of proteins as a recovery strategy has been widely used for myofibrillar and other animal proteins as well as plant proteins; usually proceeded by acid or alkaline protein solubilization (Liang and Hultin, 2003, Zhao et al., 2019; Abdollahi, Rezaei, Jafarpour, & Undeland, 2018). Isoelectric precipitation (IP) is a very fast method and the protein coagulates generated at the pI can be recovered at a relatively low centrifugation speed and do not need extra water demanding steps for removing salt residues. However, the exact pH in which the IP can take place needs to be carefully defined for each protein and source. The effect of IP on protein structure and functionality should also be studied in each specific case due to their possible effect on protein conformation. The possibility of using IP for recovery of collagen extracted from tuna skin has previously been shown (Lin et al., 2019). However, the potential of this technology for extraction of collagen from other aquatic resources and more complicated raw materials which have complex body structures such as starfish, sea urchins, sea cucumbers, lump fish etc., have not been studied. In addition, the effect of IP on structural and functional properties of collagen has not been compared side by side with SO-derived collagen which has been targeted in this study for two different resources including common starfish and lumpfish.

Shellfish producers consider *Asterias* spp. as pests and hence it is considered as one of the most destructive invasive species (Vate et al., 2022). One of the species among this, *A. rubens* causes environmental problems in northern Europe. Mussel producers often get common starfish as a by-catch while harvesting the mussels and they are usually discarded as waste although they could be valorized as a resource for collagen extraction as shown before (Han et al., 2021, Qi et al., 2017). In addition, lumpfish have been employed widely in recent years in Atlantic salmon aquaculture in sea cages as a ‘green’ environmentally friendly and cost-effective alternative to manage sea lice infestations as compared to traditional antiparasitics (Liu & Bjelland, 2014). However, the mature lumpfish stops eating lice and there is no documented potential after use once the salmon are harvested from sea cages (Ageeva, Lorentzen, Nilsen, & Lian, 2021). Skin is comprised as the major body fraction of lumpfish (Ageeva et al., 2021) which could be a potential source for collagen, something which has not been studied yet.

Bearing the mentioned drawbacks of the currently used collagen extraction methods in mind together with the existing research gaps, the present study was aimed to evaluate the possibility of using IP for collagen recovery when using two emerging marine collagen sources -starfish and lumpfish representing marine invertebrates and vertebrates, respectively. After finding the optimum pH for collagen precipitation, the effect of IP on collagen yield, structure and functional properties was also investigated and compared with the classic SO precipitation for the two targeted resources.

## 2 Material and method

### 2.1 Materials

Common starfish (*Asterias rubens*) were obtained from Scanfjord Mollösund AB (Mollösund, Sweden), a mussel farming company in Gothenburg, Sweden. They were mixed with ice and brought to the lab. Then, the starfish were cleaned with chilled water in the lab and cut into pieces of size 2.5 × 2.5 cm. Lump fish (*Cyclopterus lumpus*) were harvested from Angstauren (Troms and Finnmark county, Norway) and were transported by Nofima, Norway on dry ice to Gothenburg. The lumpfish were gutted, washed, and minced with the help of a grinder (C/E22 N, Minerva Omega group, Italy) having a plate with 3 mm holes, upon arrival in lab. Thereafter, both samples were packed separately in plastic bags and preserved at −80 °C.

### 2.2 Chemicals

The chemicals and reagents used in this study were of scientific grade. Acetic acid, sodium hydroxide, hydrochloric acid, sodium chloride and *tert*-butyl alcohol were supplied by Merck (Merck Life Sciences, Sweden). Pepsin, EDTA, bovine serum albumin (BSA), tris(hydroxymethyl)aminomethane, sodium dodecyl sulphate (SDS) and *β*-mercaptoethanol (*β*-ME) were procured from Sigma-Aldrich (USA).

### 2.3 Collagen extraction

#### 2.3.1 Pretreatment of starfish and lumpfish and collagen extraction

Frozen starfish (SF) and minced lumpfish (LF) samples were taken out from −80 °C and thawing was done under cold tap water. Before processing, thawed SF was chopped into tiny parts (0.5 to 1 cm). Both SF and LF samples were soaked in NaOH solution of 0.1 N, separately having SF or LF to solution ratio of 1:10 (w/v). The mixture was then homogenized for 2.5 min at 4000 rpm. The temperature of the sample was held below 4 °C during homogenization using an ice bath. The homogenized mixture was subjected to centrifugation at 2000 × g for 2 min. The supernatant was discarded and the precipitate was mixed with chilled water and its pH was set to 7.4. After that, it was dewatered by subjecting it to centrifugation for 5 mins at 5000 × g. The dewatered samples were subjected to demineralization using 0.5 M EDTA-2Na solution keeping the ratio of 1:15 (w/v sample to solution). The demineralization was carried out in cold room (4 °C) for 48 h by stirring with the fresh EDTA solution changed at 24 h. Residual EDTA was removed from demineralized samples by washing with the cold water. Then dewatering of the samples was done by centrifugation at 5,000 × g (5 mins at 4 °C). The pretreated starfish and lumpfish samples were used for the collagen extraction. To do so, the pretreated starfish and lumpfish samples were mixed with 0.5 M acetic acid with 1% pepsin (w/w) separately, with a sample to acid ratio of 1:15 (w/v) and stirred for 48 h at 4 °C. Then the undissolved material was separated from each sample by centrifuging for 20 min at 4 °C at a speed of 10,000 × g and the supernatant was collected for the precipitation step.

#### 2.3.2 Collagen precipitation with salting out

For salting out, the obtained supernatant from the extraction step for each sample was subjected to precipitation by adding NaCl to reach the final concentration of 2.5 M having 0.05 M of tris (hydroxymethyl) aminomethane. Then it was centrifuged at 15,000 × g for 30 min at 4 °C and the obtained precipitate was suspended in a small amount of 0.5 M acetic acid and dialyzed in 20 volumes of 0.1 M acetic acid for 48 h and subsequently in 20 volumes of distilled water for another 24 h. The dialyzed material was lyophilized using a lyophilizer (model CoolSafe 55 ScanLaf A/S, Lynge, Denmark).

#### 2.3.2 Identification of collagen isoelectric point and settings for isoelectric precipitation

To find optimum pH for precipitation of collagen, samples were taken from the extracted collagen samples of each species and their pH was set from 2 to 13 using 6 N NaOH or 6 N HCl, and centrifugation was carried out at 15,000 × g for 30 min at 4 °C. Then the protein was analyzed in the obtained supernatant using Lowry’s method (Lowry, Rosebrough, Farr, & Randall, 1951). Thereafter, solubility curve was plotted, and the isoelectric point (pI) was identified as the pH at which the collagen had the lowest solubility. The pI was integrated to the collagen recovery protocol by adjusting the pH of the extracted collagen to the pI, where after centrifugation was carried out at 15,000 × g for 30 min at 4 °C. The pellet was collected and freeze dried.

The freeze-dried collagen obtained from starfish by salting out and by isoelectric precipitation were named SFC-SO and SFC-IP respectively. Similarly, the freeze-dried collagen obtained from lumpfish by salting out and isoelectric precipitation were named as LFC-SO and LFC-IP respectively. The extracted collagen from starfish and lumpfish were used for several analysis as descried below.

### 2.4 Characterization of collagen

#### 2.4.1 Recovery and mass-yield

Recovery of collagens was measured by determining the protein content by Lowry’s method (Lowry et al., 1951) in the collagen solution before and after application the precipitation step. Recovery was depicted as percent of protein extracted considering the protein content in the collagen solution before precipitation as 100%.

The mass-yield of SF and LF collagens was calculated using the following formula:

Mass-yield (%) = (Weight of lyophilized collagen)/(Weight of initial wet raw material) × 100

#### 2.4.2 UV–visible analysis

SFC-SO, SFC-IP, LFC-SO and LFC-IP were analyzed for UV–visible spectra following the method of Duan, Zhang, Du, Yao, and Konno (2009). Collagen samples were dissolved in 0.5 M acetic acid at a concentration of 0.5 g/L and the absorbance was measured from wavelength 190 to 450 nm by means of a spectrophotometer (Cary 60 UV–vis, Agilent technologies, Santa Clara, USA).

#### 2.4.3 FT-IR analysis

FT-IR spectra of the freeze-dried samples from starfish and lumpfish was carried out using Nicolet 6700 spectrophotometer (Thermo Scientific, MA, USA). The samples were analyzed based on the method explained by Chuaychan, Benjakul, and Kishimura (2015). Collagen samples were scanned at ambient temperature (25 °C) from 4000 cm^−1^ to 400 cm^−1^ at data acquisition rate of 4 cm^−1^ per point. The spectra were collected by 32 times scanning.

#### 2.4.4 Gel electrophoresis of collagen

The protein pattern of SF and LF collagens was obtained by analyzing in SDS-PAGE following the method of Laemmli (1970) as explained by Abdollahi et al. (2018). Precast gels of 7.5 % from Bio-Rad (USA) were used to separate different protein bands. The collagen samples from starfish and lumpfish were dissolved in 5 % SDS and protein content was obtained by analyzing by using Lowry’s method (Lowry et al., 1951). Each sample with 15 μg of protein (7.5 μL) and 5 μL of marker (Bio- Rad, USA) were loaded onto the gel and electrophoresis was done at a constant current of 50 mA. Staining was done using 0.02 % (w/v) Coomassie Brilliant Blue R-250 in 50 % (v/v) methanol and 7.5 % (v/v) acetic acid followed by destaining (50 % methanol (v/v) and 7.5 % (v/v) acetic acid) for 1 h. Lastly, the gel picture was taken in Bio GelDoc Go Imaging system (Bio-Rad, USA).

#### 2.4.5 Determination of amino acid composition

Amino acid profile of the SF and LF collagens was determined according to the method described by Özcan and Şenyuva (2006) as explained by Abdollahi et al. (2018).

#### 2.4.6 Salt solubility test

Salt solubility test was carried out using the method of Jongjareonrak, Benjakul, Visessanguan, and Tanaka (2005). SFC-SO, SFC-IP, LFC-SO and LFC-IP were dissolved separately in 0.5 M acetic acid to get to the concentration of 6 mg/mL. Five mL of collagen solutions were mixed with equal amounts of cold NaCl solutions of different concentrations prepared in acetic acid, to attain the final NaCl concentrations of 1, 2, 3, 4, 5 and 6 % (w/v). The mixture was stirred mildly at 4 °C for 30 min and centrifuged at 10,000 × g at 4 °C for 30 min. The protein content in the supernatants was analyzed by the Lowry’s method (Lowry et al., 1951), and relative solubility was calculated in comparison with that found at the salt concentration exhibiting the highest solubility.

#### 2.4.7 Determination of degree of collagen fibril formation

Starfish and lumpfish collagen fibrils were formed according to the method explained by Vate et al. (2022). The collagen solutions of desired concentration were prepared in HCl and mixed with Na-phosphate buffer and stored in optimum temperature for 24 h. Then the supernatant was collected after centrifugation and analyzed for protein. The percentage reduction of protein content is considered as degree of fibril formation (DFF).

#### 2.4.8 Scanning electron microscopy of collagen

Starfish and lumpfish collagen fibrils were formed using the method explained by Vate et al. (2022) and microstructure of fibrils was carried out according to the method of Zhang et al. (2014). Alcohol dehydrated collagen fibrils were lyophilized with a freeze-drying device (model CoolSafe 55 ScanLaf A/S, Lynge, Denmark). Freeze-dried collagen fibrils were coated with gold-platinum and the microstructure was observed using a scanning electron microscope (SEM; Zeiss Ultra 55 FEG, Germany) at different magnifications.

#### 2.4.9 Thermal denaturation temperature

Thermal denaturation temperature of collagens from starfish and lumpfish were analyzed by measuring the complex viscosity using a Physica MCR300 dynamic rheometer (Paar Physica) according to the method explained by Vate et al. (2022).

### 2.5 Statistical analysis

The pretreatments and extractions of collagens were done at least twice. Analyses of the extracted collagen were run in duplicates and the significance difference was determined by subjecting the data from these analyses to analysis of variance (ANOVA). Duncan’s multiple range test was used to compare the mean values (Steel & Torrie, 1980) and the data was considered as significantly different when *p* < 0.05. Statistical Package for Social Science (IBM SPSS 28.0 for Windows, SPSS Inc., Chicago, IL, USA) was used for the statistical analysis.

## 3 Results and discussions

### 3.1 Collagen pH dependent solubility and isoelectric point

**Fig. 1:**
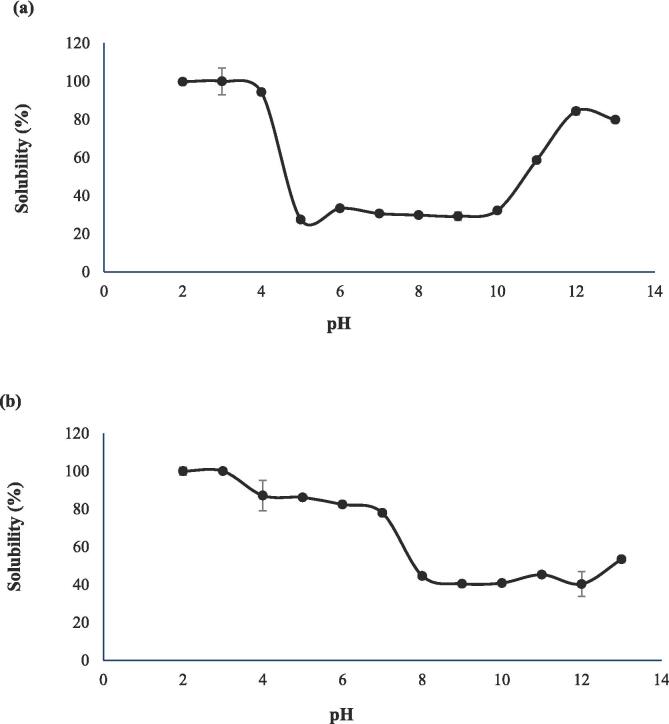
Solubility curve for starfish collagen (a) and lumpfish collagen (b) at different pH values. Data show mean values ± SD (n = 3).

The solubility of starfish and lumpfish collagen at different pH values is shown in Fig. 1. The starfish collagen sample had the highest solubility at pH 2. The solubility decreased sharply after pH 4 and showed lowest solubility at pH 5 (*p* < 0.05). The solubility increased slightly, but not significantly, at pH 6 and was stagnant until pH 10. The average solubility was significantly higher at the pH 11, 12 and 13. Since the protein has no net charge at the pI, the protein aggregates due to hydrophobic–hydrophobic interactions and cause protein precipitation. The net charge of protein increases above or below the pI increasing its interaction capacity with water (Lin et al., 2019). The result indicated that starfish collagen lacks a distinct pI, and instead, solubility was low over the whole pH range of 5–10. Therefore, pH 5 which was the pH value resulting in minimum average solubility was selected for the isoelectric precipitation of starfish collagen after pretreatment and extraction. The solubility pattern was different for lumpfish collagen compared to that of starfish collagen. Highest solubility was seen at pH 2 and 3 after which the average solubility slightly, but not significantly, decreased until pH 7. A sharp and significant decrease in solubility was observed above pH 7 and the lowest solubility was found at pH 9 and 10. The average solubility slightly (*p* > 0.05) increased at pH 13. For the precipitation of lumpfish collagen, pH 9 was selected as pI as it showed the lowest average solubility. In agreement with our results, pI-values between 6 and 9 have earlier been reported for collagen extracted from different resources such as balloon fish (*Diodon holocanthus*) and sea bass (*Lates calcarifer*) (Huang, Shiau, Chen, & Huang, 2011, Sinthusamran et al., 2013). That the solubility of collagen is lower at neutral and alkaline pH is due to the abundance of hydrophobic amino acids such as glycine, proline and alanine. The exact pH range in which collagen shows its minimum solubility will thus be dependent on its amino acid composition, which in turn is dependent on the biological source used. Both of these factors therefore likely explain the solubility differences seen between collagen from the two species. Amino acid differences are further discussed in section 3.6.

### 3.2 Mass-yield and recovery of collagen

**Fig. 2:**
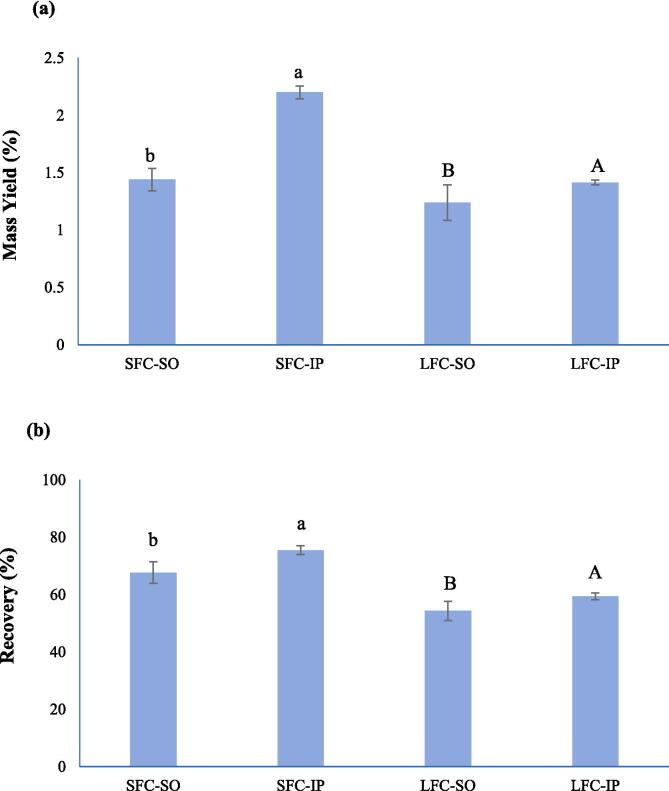
UV–visible (a) and FTIR (b) spectra of starfish and lumpfish collagens recovered with isoelectric precipitation and salting out, SFC-SO: Starfish collagen extracted by salting-out; SFC-IP: Starfish collagen extracted by isoelectric precipitation; LFC-SO: Lumpfish collagen extracted by salting-out; LFC-IP: Lumpfish collagen extracted by isoelectric precipitation.

The mass-yield and recovery of collagen isolated through SO and IP from starfish and lumpfish are summarized in Fig. 2. The mass yield of starfish collagen obtained by traditional SO method (1.44 % w/w, wet weight (ww) basis) was significantly (*p* < 0.05) lower than that obtained by IP (2.2 %). This was supported by the result from the recovery of starfish collagen which followed the same pattern. Similarly, the mass-yield of isoelectrically precipitated collagen from lumpfish (1.415 %) was higher than that obtained by salting out (1.24 %). Similar pattern was also observed in the case of recovery of lumpfish collagen by IP and SO. The protein content of SFC-SO, SFC-IP, LFC-SO and LFC-IP were 91.79 ± 4.13, 51.62 ± 4.01, 80.46 ± 0.60 and 71.14 ± 4.06 %, respectively. This indicated that the purity of collagen was higher in samples precipitated by SO. This could probably be related to removal of some non-protein compounds such as mineral residues during the dialysis-driven desalting process which is conducted after the salting out which is not included in the IP. This was supported by the ash content in the collagen samples. The collagen samples isolated by salting out method, SFC-SO and LFC-SO had lower content of ash (0.26 ± 0.17 and 0.18 ± 0.09 %, respectively) than those precipitated by IP including SFC-IP and LFC-IP having ash content of 21.57 ± 1.27 and 16.22 ± 0.80 %, respectively. Collagen mass yield from the seastar *A. amurensis* obtained by salting out was 5.8 % based on the wet tissue (Lee et al., 2009). Tan, Karim, Latiff, Gan, and Ghazali (2013) obtained a collagen mass-yield of 2.29 % (dry weight (dw) basis) from the body wall of crown-of-thorns starfish (*Acanthaster planci*). The mass yield of collagen extracted from bigeye tuna skin through IP was higher than that obtained by SO (Lin et al., 2019). However, these authors did not report the purity of the collagen recovered with IP.

### 3.3 UV-Spectra of collagens

**Fig. 3:**
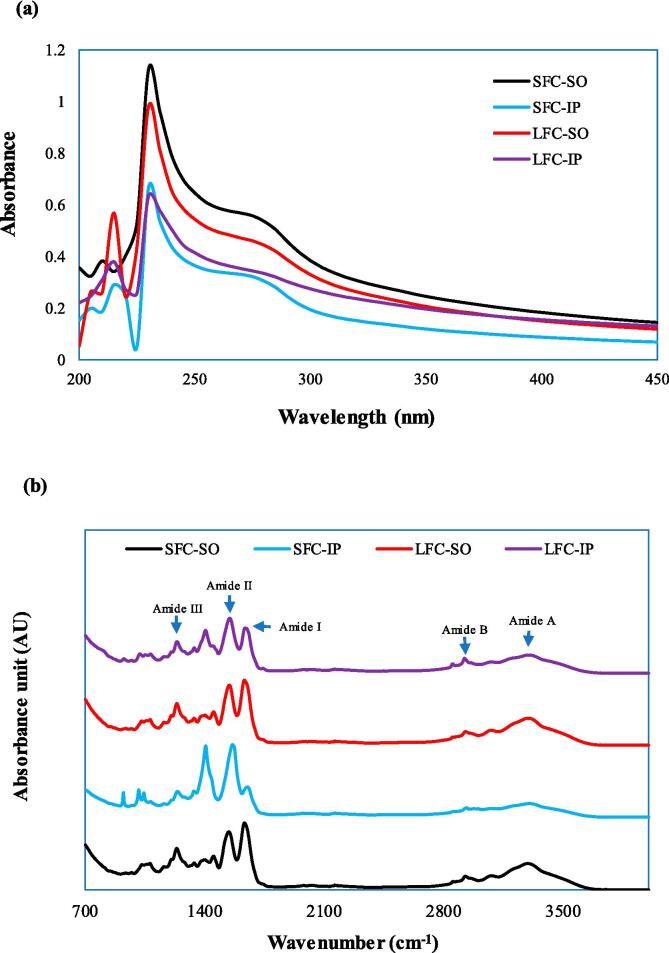
Mass yield (a) and recovery (b) of starfish and lumpfish collagens recovered with isoelectric precipitation and salting out, SFC-SO: Starfish collagen extracted by salting-out; SFC-IP: Starfish collagen extracted by isoelectric precipitation; LFC-SO: Lumpfish collagen extracted by salting-out; LFC-IP: Lumpfish collagen extracted by isoelectric precipitation.

UV- spectra of collagen samples is depicted in Fig. 3a. Pure collagen as a protein shows highest absorbance at the wavelength of 230 nm because of its triple helical structure (Pal, Nidheesh, & Suresh, 2015). Hence, the purity of collagen can be assessed by scanning it in ultra-violet (UV) spectral range. The starfish and lumpfish collagens isolated by SO and IP showed maximum absorbance at 230 nm. SFC-SO had the highest absorbance peak among all the collagen samples. The absorbance of collagen samples obtained by IP were lower than those isolated by salting out. This was most likely due to the lower content of protein in collagen extracted by IP. Collagens extracted from skin of snakehead fish exhibited their maximum absorbance at 227 and 226 nm (Liu, Zhang, Cui, & Wang, 2019). The skin collagen from Medusa fish (*Centrolophus niger*) had its highest absorbance at 232 nm (Bhuimbar, Bhagwat, & Dandge, 2019). Similar UV-spectrum was observed for skin collagens of *Catla catla* and *Labeo rohita* (Pal et al., 2015). The highest absorption obtained within the range of 220 and 240 nm indicates the occurrence of CO–NH_2_, –COOH, and C=O in the triple helical structure of the extracted collagens (Abdollahi et al., 2018). The largest absorption peaks for the collagen of sea cucumber and tilapia fish skin were observed at 236.5 and 235.5 nm respectively (Li et al., 2020). A small absorption peak was observed in collagen samples at 280 nm which is associated with the lower amounts of amino acids containing benzene rings such as phenylalanine and tyrosine in collagen which have the highest absorption at the wavelength of 280 nm (Pignataro, Herrera, & Dodero, 2020). The UV–visible spectra of starfish and lumpfish samples proved that the extracted proteins were collagen and the precipitation method did not affect their spectroscopic properties.

### 3.4 FT-IR spectra

The FT-IR spectra of SFC-SO, SFC-IP, LFC-SO and LFC -IP is demonstrated in Fig. 3b. All the collagen samples showed the distinctive peaks associated to amide bands I, II, III, A, and B. There was difference between the FTIR spectra of collagen samples prepared by SO and those prepared by IP for both starfish and lumpfish. The SO and IP precipitated collagen samples from starfish and lumpfish showed the amide bands I, II and III vibrations in the range of 1600–1700 cm^−1^, 1500–1600 cm^−1^ and 1200–1300 cm^−1^, respectively. Amide I, II and III band vibrations are characteristic of the collagen molecule (Benjakul et al., 2010). Amide I band of SFC-SO and LFC-SO had same wavelength of 1631 cm^−1^ whereas those of SFC-IP and LFC-IP were found at 1648 cm^−1^ and 1637 cm^−1^respectively. Amide I correlate the C=O stretching. The hydrogen bond establishment within the N—H part is often represented by a lower wavenumber, and the CO residue is in charge of stabilizing the triple helix structure (Zanaboni, Rossi, Onana, & Tenni, 2000). N—H bending is represented by Amide II band. SFC-IP had higher wavenumber of 1562 cm^−1^ for the amide II band than those of SFC-SO, LFC-SO and LFC-IP which had wavenumber of 1541 cm^−1^, 1543 cm^−1^ and 1547 cm^−1^, respectively. Amide I and II bands of all collagens shifted to lower wavenumber suggesting more hydrogen bonds were formed in the triple helix. In case of amide III band, both SFC-SO and LFC-SO showed wavenumber of 1236 cm^−1^. This indicated that hydrogen bonds were involved in preserving the native structure. SFC-IP and LFC-IP had amide III band at the wavenumbers of 1240 cm^−1^ and 1238 cm^−1^, respectively. Amide III represents the C—N stretching and N—H deformation, which are involved in the complex intermolecular interactions in collagen (Sinthusamran et al., 2013). The intensity of amide I band in SFC-IP was much lower than that of SFC-SO. On the other hand, the band intensity of amide II was higher in SFC-IP compared to the other collagen samples. The result suggested that the acidic pH used for the IP affected the structure of starfish collagen. This could also be related to the difference in the pH of the two samples during drying affecting their structure. Also, there was a high intensity band observed in SFC-IP in the wavenumber range of 1400 cm^−1^. This corresponds to the COO-symmetrical stretch (Pal et al., 2015). The FTIR spectra of collagens, specially SFC-SO and LFC-SO were similar to that of sea cucumber (Li et al., 2020) and collagens from skin of seabass (Sinthusamran et al., 2013) and channel catfish (Liu, Li, & Guo, 2007).

The range of absorption peak for Amide A is between 3400 and 3440 cm^−1^ (Doyle, Blout, & Bendit, 1975). The amide A band of SFC-SO, SFC-IP, LFC-SO and LFC-IP was found at the wavenumbers of 3292, 3305, 3294 and 3296 cm^−1^, respectively. The N—H stretching vibration is indicated by the Amid A band. The intensity of amide A band of collagen samples isolated by IP were lower than those extracted by SO. It was inferred from the wavenumber's change to a lower frequency that the NH group was engaged in hydrogen bonding (Benjakul et al., 2010). The amide B band is linked to the asymmetrical stretching of CH_2_ and it was found at the same wavenumber of 3078 cm^−1^ for all collagen samples. The triple helix structure of collagen can be confirmed by ratio of band intensity between amide III and 1450 cm^−1^ bands (Benjakul et al., 2010) which was about 1.1 for all the collagens in the present study. This confirmed that all collagen samples from starfish and lumpfish preserved the native triple helix which is essential for its application in biomedical field. The acidic pH used for precipitation of starfish collagen had milder effect on triple helical structure especially in the area where structure is stabilized by C=O and COO. Overall, the FT-IR spectral analysis revealed that the triple helical structure of all the collagen were preserved despite the different precipitation methods.

### 3.5 Protein pattern

**Fig. 4:**
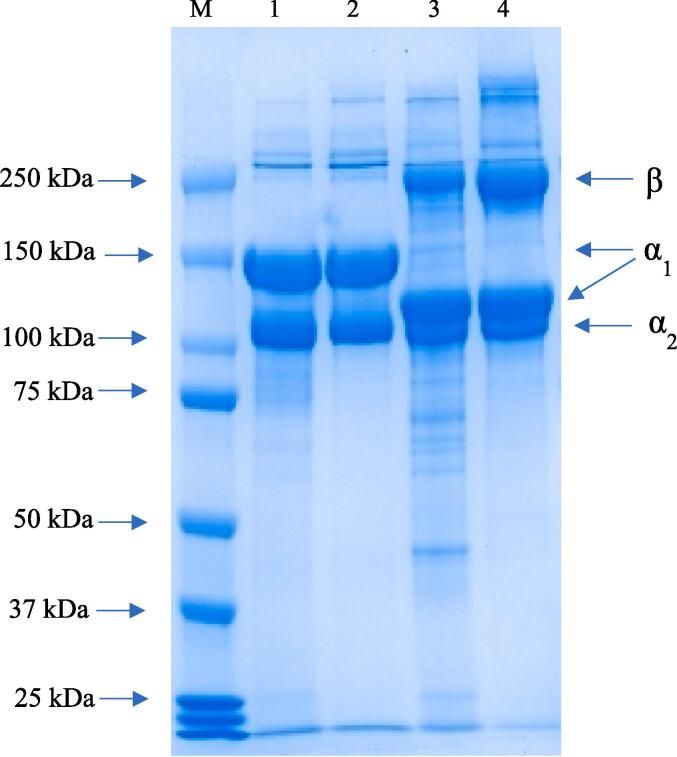
Polypeptide patterns of collagen samples extracted from starfish and lumpfish followed by recovery with isoelectric precipitation and salting out, M: Marker; 1: Starfish collagen extracted by salting-out; 2: Starfish collagen extracted by isoelectric precipitation; 3: Lumpfish collagen extracted by salting-out; 4: Lumpfish collagen extracted by isoelectric precipitation.

The electrophoresis pattern of collagen from starfish and lumpfish precipitated by SO and IP is shown in Fig. 4. The polypeptide patterns showed the distinctive bands correlating to the collagen. The molecular weight of protein bands of collagens extracted by SO were similar to that extracted by IP in both starfish and lumpfish. However, the polypeptide patterns of starfish collagens were different than that of lumpfish collagen. Both starfish and lumpfish collagens isolated by SO and IP had α and β chains. The α_1_ chain of starfish collagens had higher molecular weight than that of lumpfish collagens. Contrary, the β chain band intensity of lumpfish collagens was higher than that of the starfish collagens. This indicated that there was a difference between the structure of collagen extracted from starfish and lumpfish with more intermolecular crosslinking existing in lumpfish collagen. This is most likely due to the structure of the body wall of starfish versus that of skin and bones of lumpfish from which collagen was extracted. The ratio between the α_1_ to α_2_ chains in collagen samples isolated using SO and IP from both starfish and lumpfish collagens was 2:1 indicating that the collagen from both organisms were of type I. The polypeptide pattern of starfish collagen was similar to that for collagen from the seastar *A. amurensis* having a structure similar to type I collagen with α1, α2 and β- bands (Lee, Park, Kim, Park, & Yoon, 2009). Our SDS-PAGE result of starfish collagen was also similar to the polypeptide patterns of pepsin soluble collagen (PSC) from the body wall of crown-of-thorns starfish (*Acanthaster planci*) (Tan et al., 2013). The SDS-PAGE result of lumpfish collagen was comparable to polypeptide pattern of collagen from skin of fishes such as paddle fish (Wang, Sun, & Zhou, 2017), golden carp (Ali, Kishimura, & Benjakul, 2018) and channel catfish (Tan & Chang, 2018). Also, the result showed that the polypeptide pattern of collagen samples was not affected by the type of precipitation method.

### 3.6 Amino acid content of the collagen

Amino acid content of the collagens extracted from starfish and lumpfish obtained by SO and IP is shown in Table 1 (Supplementary). In all the collagens from starfish and lumpfish the amino acid glycine was found in highest quantity irrespective of the precipitation methods. This is consistent with the distinctive amino acid sequence for collagen, in which glycine is found at every third amino acid residue (Kittiphattanabawon, Benjakul, Visessanguan, Nagai, & Tanaka, 2005). The amount of glycine in the collagen samples recovered with SO or IP did not differ significantly. Starfish collagens isolated by both SO and IP had glutamic acid (Glu) as the second most dominant amino acid while proline (Pro) was the second major amino acid in lumpfish collagen samples. The amount of lysine was higher in lumpfish collagen compared to that in starfish collagen. Lysine is one of the major amino acids found in fish proteins as a whole (Ozden, 2005). The amino acid profile of collagen subunits is a repeated chain of Glycine-X-Y, where X and Y are variables, although they are usually occupied by proline and hydroxyproline, respectively (Abdollahi et al., 2018). The dominance of glycine in starfish collagen was in agreement with the amount of glycine described for collagen extracted from the body wall of *Acanthaster planci* (Tan et al., 2013) and the entire amino acid profile of the collagens extracted from starfish was comparable to that found for collagen from seastar *A. amurensis* (Lee et al., 2009) and from purple sea urchin (*Anthocidaris crassispina*) (Nagai & Suzuki, 1999). Collagen peptides from *A. pectinifera* also had glycine as the main amino acid (Han et al., 2021). The relatively higher amount of glycine-proline which, according to above, are characteristic for collagen, indicated that collagen was the main content of the extracts recovered by SO and IP from starfish and lumpfish. Overall, the results showed that by replacing the classic SO collagen precipitation method with IP, the amino acid profile of collagens recovered from starfish and lumpfish is not affected.

### 3.7 Thermal denaturation temperature of collagen

**Fig. 5:**
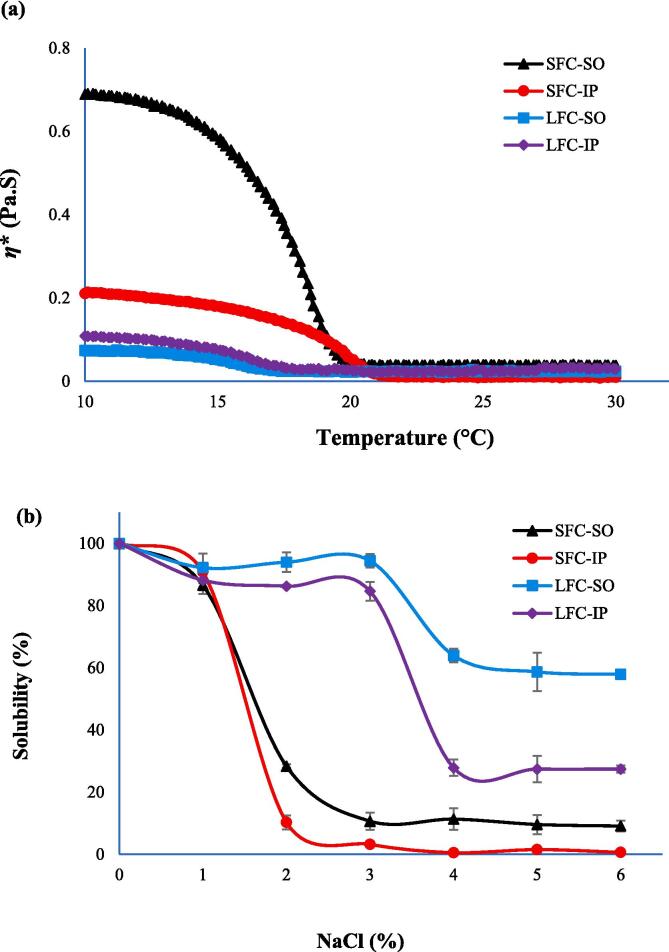
Temperature sweep (a) and salt dependent solubility (b) of starfish and lumpfish collagens recovered with isoelectric precipitation and salting out, SFC-SO: Starfish collagen extracted by salting-out; SFC-IP: Starfish collagen extracted by isoelectric precipitation; LFC-SO: Lumpfish collagen extracted by salting-out; LFC-IP: Lumpfish collagen extracted by isoelectric precipitation.

The thermal denaturation of the collagen samples was analyzed by determining the changes in complex viscosity (Fig. 5a). The complex viscosity of all the collagen samples dropped with the temperature rise regardless of the precipitation method. This drop in viscosity is primarily result of lowered hindrance to the segment motion caused by rise in the energy for the thermal movement of protein chains (Zhang, Chen, Li, & Du, 2010). SFC-SO had the highest initial viscosity compared to other collagen samples. This was most likely explained by the fact that it had the highest protein content. The complex viscosity of SFC-SO reached its lowest value at the temperature around 21 °C and stabilized thereafter till 30 °C. The complex viscosity of SFC-IP continued to decrease until the temperature reached around 22 °C, after which it became stagnant. The lumpfish collagen samples obtained both by SO and IP reached their lowest complex viscosity around the temperature of 19 °C. The lowering of η* in magnitudes indicated the denaturation of the collagen triple helix into a random coil. The temperature at which the decrease of η* reaches to 50% of the initial value is considered as the denaturation temperature (Lai, Li, & Li, 2008). SFC-IP had the highest denaturation temperature of 18.9 °C followed by SFC-SO having the denaturation temperature of 17.6 °C (*p* < 0.05). The lumpfish collagen samples obtained by SO and IP had similar denaturation temperatures of 16.1 and 16.2 °C, respectively. The result indicated that the use of different precipitation methods did not have any negative effect on the thermal denaturation of the starfish and lumpfish collagens. Collagen from seastar, *A. amurensis* had the thermal denaturation temperature of 24.7 °C based on drop in complex viscosity (Lee et al., 2009). Similar findings have been reported for type I collagen extracted from skin of *Mystus macropterus* (Zhang et al., 2010). The thermal property of collagens mostly relies on their imino acid content such as proline. It is also associated with the temperatures of the body of the organism from which collagen is extracted from along with temperatures of environment in which they live in Minh Thuy, Okazaki, and Osaka (2014). The denaturation temperature of marine collagen isolated from cold-water species is substantially lower than that of warm-water species (Jafari et al., 2020). The denaturation temperatures of collagens from starfish and lumpfish obtained in present study are closer to those described for other cold-water species like cod skin (14.5 °C) and salmon skin (19 °C) (Sun, Li, Song, Si, & Hou, 2017, Yunoki, Suzuki, & Takai, 2003).

### 3.8 Salt solubility

NaCl solubility of collagens recovered by SO and IP from starfish and lumpfish is depicted in Fig. 5b. Within the same raw material, the collagen obtained by IP had lower salt solubility than those isolated by SO (*p* < 0.05). The solubility of starfish collagens was maintained up until 2 % NaCl. These collagens reached their lowest solubility at a NaCl concentration of 3% and remained low until 6 % NaCl. The higher solubility of lumpfish collagens continued until a NaCl concentrations of 3 % was reached, and these collagens had their lowest solubility at 4, 5 and 6 % NaCl. SFC-IP had the lowest solubility and LFC-SO the highest solubility among all the samples. The lower solubility of collagen samples at higher NaCl concentrations reflects the ‘salting-out’ phenomenon caused by increased hydrophobic-hydrophobic interaction along with interchain polymerization (Hukmi & Sarbon, 2018). The interaction between protein chains increases at increased ionic strength resulting from Na^+^ and Cl^-^ ions competing for water with the proteins, resulting in protein precipitation (Tan & Chang, 2018). NaCl solubility of starfish collagens were comparable with that of PSC from the seastar *A. amurensis* which dropped at salt concentration of >3% (Lee et al., 2009). The collagen isolated from body wall of seastar *Asterina pectinifera* were also less soluble at higher NaCl concentrations (Qi et al., 2017). Similar NaCl solubility patterns between collagen extracted by SO and IP indicated that there was no negative effect caused on collagen quality by the modifications in the precipitation process.

### 3.9 Degree of fibril formation

**Fig. 6:**
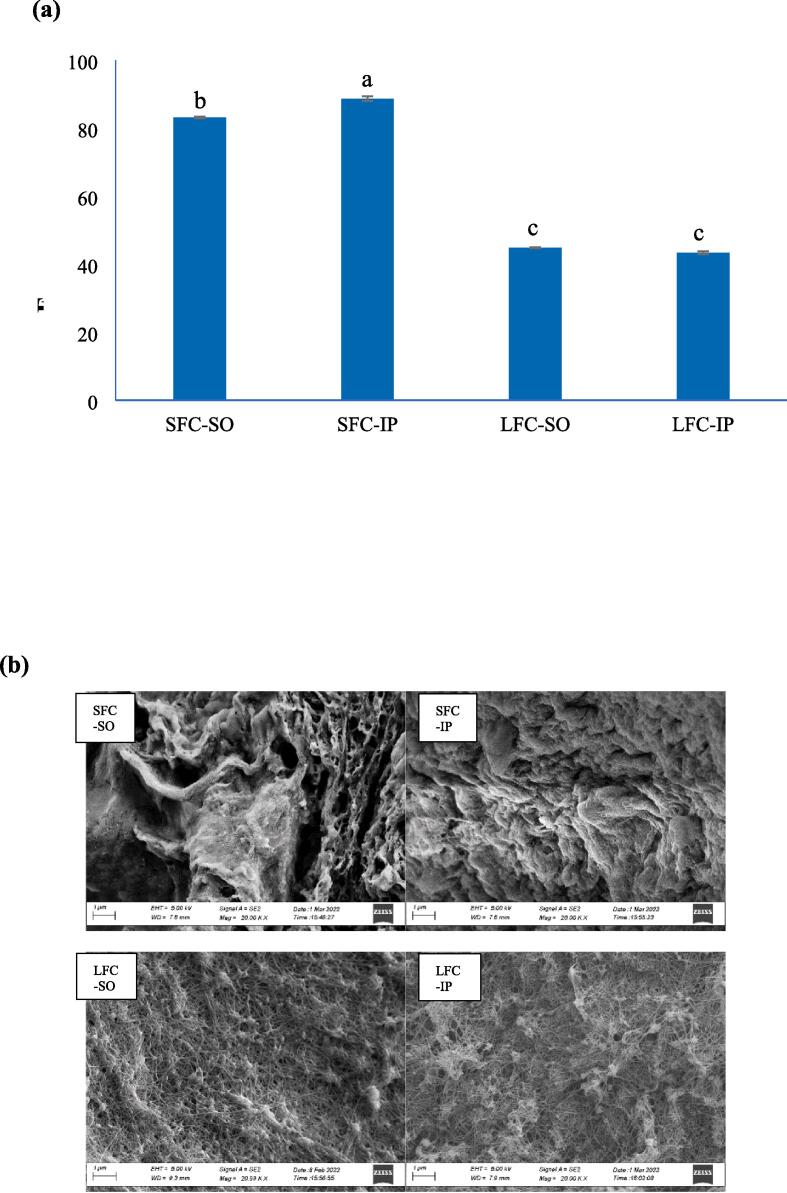
Degree of fibril formation (a) and SEM images (b) of fibrils from starfish and lumpfish collagens recovered with isoelectric precipitation and salting out, SFC-SO: Starfish collagen extracted by salting-out; SFC-IP: Starfish collagen extracted by isoelectric precipitation; LFC-SO: Lumpfish collagen extracted by salting-out; LFC-IP: Lumpfish collagen extracted by isoelectric precipitation.

The starfish and lumpfish collagen samples showed fibril formation capacity as evidenced by the DFF (Fig. 6a). Within starfish collagens the collagen isolated by IP had the highest DFF compared to the one precipitated with the aid of SO. This is most likely due to the presence of more polar amino acids such as tyrosine in SFC-IP compared to SFC-SO as shown in the amino acid composition of the sample (see Table 1, Supplementary). No significant difference was observed in DFF values for lumpfish collagens precipitated by SO and IP. The DFF was higher for collagen extracted from swim bladder of Bester sturgeon than that of skin collagen from same fish, and also compared to porcine collagen (Zhang et al., 2014). This indicated that the DFF of collagen depends on the resource it is extracted from. The structure of the body wall is much different from the structure of lumpfish skin and body. Hence the collagen extracted from starfish collagens had higher DFF than those from lumpfish. However, the different precipitation techniques did not affect the DFF meaning the functionality of the collagens is not affected by replacing the SO method with the IP method. The DFF was comparable to that found for collagen from skin of river puffer and tiger puffer (Wang, Yu, Sun, Liu, & Zhou, 2019).

### 3.10 Scanning electron micrographs (SEM) of collagen fibrils

The SEM images of collagen fibrils isolated by SO and IP from starfish and lumpfish collagen are shown in Fig. 6b. The fibrils from starfish collagens, both SFC-SO and SFC-IP, showed a very compact and dense structure. This rendered the observation of individual fibers from starfish collagen almost impossible. The thicker structure could suggest that more collagen proteins were involved in the fibril formation for starfish. Among fibrils from starfish collagens obtained by SO and IP, the SFC-IP collagen fibril had more compact structure than SFC-SO. This was in accordance with result of degree of fibril formation which was 83% vs 88% for collagen recovered with SO and IP, respectively. The fibrils from lumpfish collagen were thinner with interconnections, which made observation of the individual fibers possible. They were less compact and more fibrous, including some voids. There was no difference observed in the structure of fibril from lumpfish collagens between the two isolation methods. This was also indicated by the degree of fibril formation values. The fibrils from lumpfish collagen showed interconnected network structure even though they showed lower degree of fibril formation. The microstructure revealed that there could be formation of fibrils even though there was a lower degree of fibril formation and can have different structures based on the type of raw material that the collagen has been extracted from. Also, it showed that the precipitation method has minor effects on the fibril structures. The microstructure of collagen from lumpfish was similar to that found for collagens from skin and swim bladder of sturgeon (Zhang et al., 2014) and from skin of river puffer and tiger puffer (Wang, Yu, Sun, Liu, & Zhou, 2019).

## 4 Conclusions

Collagens were successfully extracted from starfish and lumpfish by replacing salting-out during the precipitation step with isoelectric precipitation. pH 5 and 9 were identified as optimum pH values for isoelectric precipitation of collagen from starfish and lumpfish, respectively. Isoelectric precipitation resulted in higher collagen mass yield and recovery from starfish and lumpfish compared with salting-out. However, collagens extracted with isoelectric precipitation showed lower purity compared with those recovered with salting-out. SDS-PAGE result indicated that the extracted collagens from both starfish and lumpfish were of type-I and there was no difference between the polypeptide pattern of the salting-out recovered and isoelectric precipitated collagens. FT-IR spectra showed that the triple helical structure of collagen was well conserved for collagens isolated by both precipitation methods from starfish and lumpfish. Starfish collagens had higher denaturation temperature and fibril forming capacity than lumpfish collagens but the functionality of collagens from these two species was not affected by replacing salting-out with isoelectric precipitation. Altogether, the results indicated that isoelectric precipitation can be an effective alternative precipitation method after the collagen extraction step. This can substantially reduce the time and chemicals required during the collagen extraction.

## CRediT authorship contribution statement

**Naveen Kumar Vate:** Data curation, Investigation, Methodology, Visualization, Writing – original draft. **Przemyslaw Pawel Strachowski:** Data curation, Investigation, Methodology. **Ingrid Undeland:** Writing – review & editing, Funding acquisition, Project administration. **Mehdi Abdollahi:** Conceptualization, Supervision, Writing – review & editing, Funding acquisition, Project administration.

## Declaration of Competing Interest

The authors declare that they have no known competing financial interests or personal relationships that could have appeared to influence the work reported in this paper.

## Data Availability

Data will be made available on request.
