# Supplementary material for: Structural and functional properties of collagen isolated from lumpfish and starfish using isoelectric precipitation vs salting out
Source: Food Chem X. 2023 Mar 15;18:100646. doi: 10.1016/j.fochx.2023.100646 (PMC10053375; doi:10.1016/j.fochx.2023.100646)
Supplement: Supplementary data 1 [file mmc1.docx]

**Supplementary Table 1**: Amino acid composition of collagen samples from starfish and lumpfish (mg/g protein)

| AminoAcid/Sample | SFC-SO | SFC-IP | LFC-SO | LFC-IP |
| --- | --- | --- | --- | --- |
| Lysine | 18.39±1.52^c^ | 19.32±2.69^c^ | 31.50±0.90^a^ | 25.99±3.14^b^ |
| Arginine | 64.06±7.37^a^ | 36.88±8.86^c^ | 59.62±4.33^b^ | 40.30±8.84^c^ |
| Histidine | 3.41±0.39^b^ | 1.22±0.51^c^ | 6.31±0.16^a^ | 3.33±0.95^b^ |
| Glycine | 173.51±12.10^a^ | 162.43±22.98^a^ | 190.76±6.44^a^ | 164.19±19.77^a^ |
| Alanine | 62.73±3.15^a^ | 60.94±7.26^a^ | 61.90±1.89^a^ | 54.76±7.91^a^ |
| Serine | 62.24±2.79^a^ | 58.55±9.23^ab^ | 58.48±1.30^ab^ | 50.19±5.79^b^ |
| Threonine | 26.33±2.12^ab^ | 26.51±3.35^ab^ | 26.85±0.93^a^ | 21.91±2.40^b^ |
| Glutamine | 104.88±8.65^a^ | 100.03±14.12^a^ | 90.15±1.77^ab^ | 72.66±9.56^b^ |
| Aspargine | 66.41±3.31^a^ | 64.17±7.34^ab^ | 56.26±1.35^b^ | 46.28±5.98^c^ |
| Proline | 83.85±2.24^a^ | 86.43±11.99^a^ | 92.52±2.78^a^ | 78.75±9.41^a^ |
| Valine | 23.69±1.63^ab^ | 25.27±2.52^a^ | 23.99±0.15^ab^ | 20.37±2.32^c^ |
| Methionine | 4.72±2.78^a^ | 5.87±1.30^a^ | 4.01±0.20^a^ | 4.11±0.16^a^ |
| Tyrosine | 11.98±0.79^b^ | 18.41±1.04^a^ | 11.47±0.20^b^ | 12.03±0.13^b^ |
| Isoleucine | 19.51±1.59^a^ | 20.74±2.06^a^ | 12.42±0.25^b^ | 9.63±0.97^c^ |
| Leucine | 21.21±1.78^b^ | 22.97±1.99^ab^ | 25.20±0.23^a^ | 19.82±1.84^b^ |
| Phenylalanine | 8.78±0.85^c^ | 10.57±0.59^c^ | 17.91±0.22^a^ | 15.32±1.59^b^ |

SFC-SO: Starfish collagen extracted by salting-out; SFC-IP: Starfish collagen extracted by isoelectric precipitation; LFC-SO: Lumpfish collagen extracted by salting-out; LFC-IP: Lumpfish collagen extracted by isoelectric precipitation

*Results are given as mean±S.D (*n*=3). Different superscripts in the same row indicate significant differences (*p<*0.05).
